# Metagenomic Analysis of Antibiotic Resistance Genes in Untreated Wastewater From Three Different Hospitals

**DOI:** 10.3389/fmicb.2021.709051

**Published:** 2021-08-24

**Authors:** Xiurong Guo, Nan Tang, Hui Lei, Qi Fang, Li Liu, Quan Zhou, Can Song

**Affiliations:** School of Pharmacy, Southwest Medical University, Luzhou, China

**Keywords:** metagenomic, antibiotic resistance genes, hospital wastewater, microbiota, bacterial pathogens

## Abstract

Controlling antibiotic resistance genes (ARGs) is a worldwide intervention to ensure global health. Hospital wastewater is the main pollution source of antibiotic-resistant bacteria and ARGs in the environment. Expanding our knowledge on the bacterial composition of hospital wastewater could help us to control infections in hospitals and decrease pathogen release into the environment. In this study, a high-throughput sequencing-based metagenomic approach was applied to investigate the community composition of bacteria and ARGs in untreated wastewater from three different types of hospitals [the general hospital, traditional Chinese medicine (TCM) hospital, and stomatology hospital]. In total, 130 phyla and 2,554 genera were identified from the microbiota of the wastewaters, with significantly different bacterial community compositions among the three hospitals. Total ARG analysis using the Antibiotic Resistance Genes Database (ARDB) and Comprehensive Antibiotic Resistance Database (CARD) revealed that the microbiota in the wastewaters from the three hospitals harbored different types and percentage of ARGs, and their composition was specific to the hospital type based on the correlation analysis between species and ARG abundance, some ARGs contributed to different bacterial genera with various relationships in different hospitals. In summary, our findings demonstrated a widespread occurrence of ARGs and ARG-harboring microbiota in untreated wastewaters of different hospitals, suggesting that protection measures should be applied to prevent human infections. Concurrently, hospital wastewater should be treated more specifically for the removal of pathogens before its discharge into the urban sewage system.

## Introduction

Antibiotic resistance poses a serious challenge to the treatment of pathogenic infections. However, antibiotic resistance genes (ARGs) are not only the outcome from human clinic settings, but it can also come from the interaction with animals, plants, soil, sea, and environmental samples. Two-thirds of antibiotics are consumed by animal husbandry (Done et al., 2015), and some were used in crops (Taylor and Reeder, 2020), which increase the ARGs in the environment. This may be relevant to human health interventions on food, water, and sewage. Except for the precision medication in the clinic with antibiotics, the controlling of ARGs need the social system’s efforts in the whole world (Zinsstag et al., 2011; Hernando-Amado et al., 2020). Since the COVID-19 outbreak, people from most countries are still being newly infected, which is recognized in the microbiology world as the concept of all humans sharing “One Health” (Ruckert et al., 2020). As most antibiotic resistance (AR) pathogens are released into the natural ecosystems by humans and animals (Karkman et al., 2019), analysis of ARGs in wastewater from hospitals, farms, and wastewater treatment plants is essential (Karkman et al., 2018; Manyi-Loh et al., 2018; Kayali and Icgen, 2020). In hospital wastewater, the levels of AR are different from those in the natural environment (Rodriguez-Mozaz et al., 2015). Hospital effluents are a mixture of different compounds, including pharmaceuticals, diagnostic agents, disinfectants, and metabolites of these compounds. They are highly hazardous because of their infection rate and toxicity. These wastewaters should be treated to reduce transmission of antibiotic resistance bacteria (ARB) to the ecosystem, which is one type of intervention to control resistance (Petrovich et al., 2020). Another intervention is controlling antimicrobials use, such as selecting novel antimicrobials with limited capacity to ARBs or using fewer antimicrobials. Moreover, traditional Chinese medicines (TCMs) were used in the treatment of infectious diseases to avoid antibiotic resistance (Cai et al., 2017; Su et al., 2020).

Some studies have shown a correlation between antibiotics, ARGs, and antibiotic-resistant bacteria in hospital wastewaters (Lira et al., 2020; Lutterbeck et al., 2020; Baraka et al., 2021). There were positive correlations between selected ARGs (sul1, sul2, tetQ, and qnrS) and the concentrations of certain antibiotics in the wastewaters of five hospitals in Xinjiang, China (Li et al., 2016). There were also correlations between antimicrobial residues and bacterial populations as well as between the prevalence of ARGs and bacterial populations in a wastewater treatment plant system of an urban hospital (Varela et al., 2014). However, the types and concentrations of antibiotics in hospital effluents vary. The categories of drugs administered and the duration of administration also vary depending on the type of hospital. In addition, the consumption of antibiotics was found to be seasonally dependent in one city, with no correlation between the seasonal consumption of antibiotics and the total levels of antibiotics in the city’s wastewater (Coutu et al., 2013). By examined hospital effluents from four different types of hospitals (university hospital, general hospital, pediatric hospital, and maternity hospital) and the amount of pharmaceuticals in wastewater varied according to their scale (Santos et al., 2013). In fact, antibiotic usage in the treatment of different clinical departments should vary. For the treatment of odontogenic infections, common antibiotics such as amoxicillin, amoxicillin-clavulanic acid, clindamycin, azithromycin ciprofloxacin, metronidazole, gentamycin, and penicillin are used (Poveda-Roda et al., 2007). However, for the primary health sector, the drugs with high concentrations in wastewater were furosemide, ibuprofen, oxytetracycline, and ciprofloxacin (Stuer-Lauridsen et al., 2000). Thus, these factors may lead to differences in bacterial populations and ARG prevalence in wastewaters.

In this study, we evaluated the wastewaters of three hospitals affiliated with Southwest Medical University: Affiliated Hospital of Southwest Medical University (A group), Affiliated Hospital of TCM (B group), and Affiliated Hospital of Stomatology (C group). As drug usage and treatment regimens were dependent on the diseases being treated and the specialists at each hospital, the bacterial populations were expected to be affected, which would result in a significant difference in the prevalence of ARGs in the microbiome. Thus, we aimed to identify the bacterial community composition and prevalence of ARGs in the wastewaters from these three hospitals using high-throughput sequencing analysis. In addition, we attempted to determine special interventions for the protection against infections and the pretreatment of wastewater before its discharge into sewers, which may help reduce ARGs.

## Materials and Methods

### Sample Collection

From each hospital (A, B, and C groups), 500ml wastewater was collected on October 12, 21, and 30, 2020, that is total nine samples were collected from the outflow of daily medical applications and stored in sterile 500ml glass bottles. Microbial samples from the wastewater were collected by filtration using filter membranes (0.2μm in diameter) and stored in sterilized centrifuge tubes at −80°C. Then, the filter membranes were sent to Majorbio Bio-Pharm Technology Co., Ltd. (Shanghai, China) for DNA extraction and Illumina HiSeq (pair-end library) sequencing.

### DNA Extraction and ARG Detection

Total DNA was extracted from the triplet wastewater samples of the three groups using filter membranes with the MP Fast DNA™ Spin Kit for Soil according to the manufacturer’s instructions. The concentration of the extracted DNA was determined by spectrophotometry (TBS-380 followed by NanoDrop 2000). The DNA extract quality was assessed using 1% agarose gel electrophoresis.

Genomic DNA was fragmented to 300bp (average size) using Covaris M220 (Gene Company Limited, China), and a sequencing library was prepared using NEXTFLEX Rapid DNA-Seq Kit (Bioo Scientific, Austin, TX, United States). Sequencing was performed using Illumina MiSeq (Illumina Inc., San Diego, CA, United States) according to the manufacturer’s instructions. Sequence data associated with this project have been deposited in the National Center for Biotechnology Information (NCBI) Short Read Archive database (accession no. PRJNA723368).

### Sequence Quality Control and Genome Assembly

SeqPrep was used to merge paired reads from the 3' and 5' ends.^1^ Low-quality reads (length<50bp, quality value<20, or containing N bases) were removed using Sickle.^2^ Metagenomics data were assembled using MEGAHIT (Li et al., 2015) with succinct de Bruijn graphs.^3^ A contig with a length of 300bp or more was selected as the final assembling result and used for further gene prediction and annotation.

### Gene Prediction, Taxonomy, and Functional Annotation

Open reading frames (ORFs) from each assembled contig were predicted using MetaGene (Noguchi et al., 2006). Of these predicted ORFs, lengths of over 100bp were retrieved and translated into amino acid sequences using the NCBI translation table.^5^

We clustered all predicted genes with 95% sequence identity (90% coverage) using CD-HIT (Fu et al., 2012). Among the clusters, the longest sequences were selected as representative sequences to construct a non-redundant (NR) gene catalog. For all samples, the reads with quality control were mapped to the representative sequences with 95% identity using SOAPaligner (Li et al., 2008).^6^ The gene abundance was then evaluated.

### Data Analysis

The NCBI NR database was used to align the representative sequences of the NR gene catalog for taxonomic annotations, with parameter *e*-values ≤1e-5 using BLASTP (Version 2.2.28+; Altschul et al., 1997).^7^ BLASTP against the evolutionary genealogy of genes, Non-supervised Orthologous Groups (eggNOG) database (Version 4.5, *e*-value cutoff of 1e^−5^), was used for annotating a cluster of orthologous groups of proteins (COG; Tatusov et al., 2003; Jensen et al., 2008). BLASTP against the Kyoto Encyclopedia of Genes and Genomes (KEGG) database (Xie et al., 2011; *e*-value cutoff of 1e-5) was used for KEGG annotation.^8^ BLASTP against the Antibiotic Resistance Genes Database (ARDB)^9^ and Comprehensive Antibiotic Resistance Database (CARD)^10^ was used for antibiotic resistance gene annotation (*e*-value cutoff of 1e-5). The sequence identity was ≥90%, and the alignment length was ≥30 amino acids. Other analyses were performed using Cloud Majorbio.^11^ Kruskal-Wallis H test, FDR correction, and Tukey-Kramer test were used to analyze the differences among multiple groups. Hierarchical clustering and principal coordinate analysis (PCoA) were also performed with a Bray-Curtis distance matrix using the R software package. A value of *p*<0.05 was considered statistically significant. A co-occurrence network was employed to visualize the correlation between antibiotic types and microbial taxa. A connection indicated a strong (*ρ*>0.5) and significant (value of *p*<0.05) Spearman’s correlation.

## Results

### Overview of Assembly and Annotation

In total, 847, 368, 612 clean reads were generated, with an average of 94, 152, 068 reads per sample. The statistical information of the contigs is listed in Supplementary Table S1. For each DNA dataset, annotation of the protein-coding genes was performed using a BLASTP search against the eggNOG database. Metagenomic assembly, annotations, and predicted ORFs are listed in Supplementary Table S2. The gene sequences predicted by the samples were clustered using CD-HIT to construct the NR gene catalog, and the base sequences of the genes in the non-redundant gene catalog were obtained; details are listed in Supplementary Table S3.

### Bacterial Community Characteristics in Wastewaters From Three Different Hospitals

**Figure 1:**
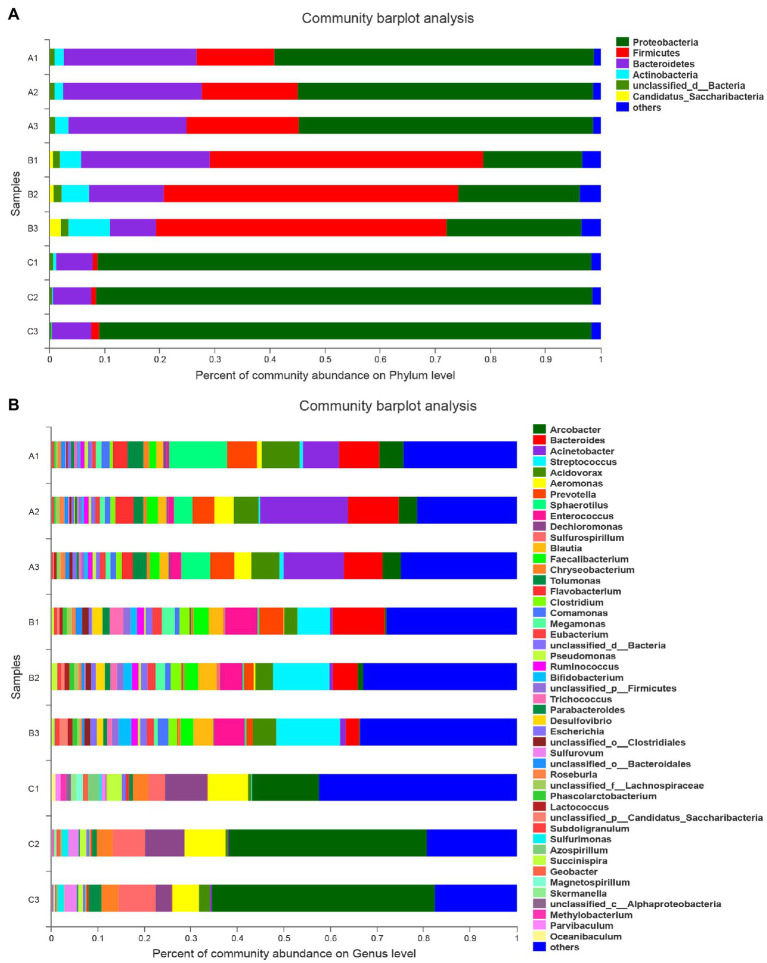
Percent of community abundance of microbiota on Phylum and Genus level in wastewater from hospitals. **(A)** Percent of community abundance on Phylum level; **(B)** Percent of community abundance on Genus level. A group (A1, A2, and A3): wastewater from Affiliated Hospital of Southwest Medical University, B group (B1, B2, and B3): wastewater from Affiliated Traditional Chinese Medicine (TCM) Hospital of Southwest Medical University, C group (C1, C2, and C3): wastewater from Hospital of Stomatology Southwest Medical University.

The bacterial community composition in the wastewaters was determined by the corresponding species and their taxonomic annotation information compared with the NCBI NR database. As shown in Figure 1A, there was no difference between the three samples of wastewater collected on different days from each group at the phylum level, indicating that these samples are generally representative of the community abundance of bacteria in the wastewater.

In total, 130 phyla and 2,554 genera were identified from the microbiota of the wastewaters. When comparing the community abundance of bacteria at the phylum level in the wastewaters of the different hospitals, Proteobacteria was the dominant phylum in the C group (89.35–90.03%), while the A and B groups exhibited a relatively low abundance of this phylum (53.42–58.00 and 18.07–24.44%, respectively). For the B group, Firmicutes were the most abundant phylum (49.58–53.44%), while the abundance of this phylum was low in both A and C groups (14.20–20.44 and 0.87–1.44%, respectively). When evaluating data at the genus level, the composition of the microbiota was also different between the three groups (Figure 1B). The most abundant genus was *Acinetobacter* (7.80–18.87%) in the A group, *Streptococcus* (6.98–13.78%) in the B group, and *Arcobacter* (14.28–47.90%) in the C group.

There was a significant difference at the phylum and genus levels between the hospitals. Samples from the A and B groups were from general hospitals that treated similar diseases but used different therapeutic regimens and drugs. Thus, these factors resulted in similar bacteria in the wastewaters but significantly different community compositions. Moreover, comparing samples from the A and B groups with the ones from the C group, a significant difference in community composition was observed. The C group hospital specializes in oral diseases, and the pharmaceuticals used for treatments here led to a less diverse bacterial community in the wastewater compared with the bacterial communities of the two general hospitals.

**Figure 2:**
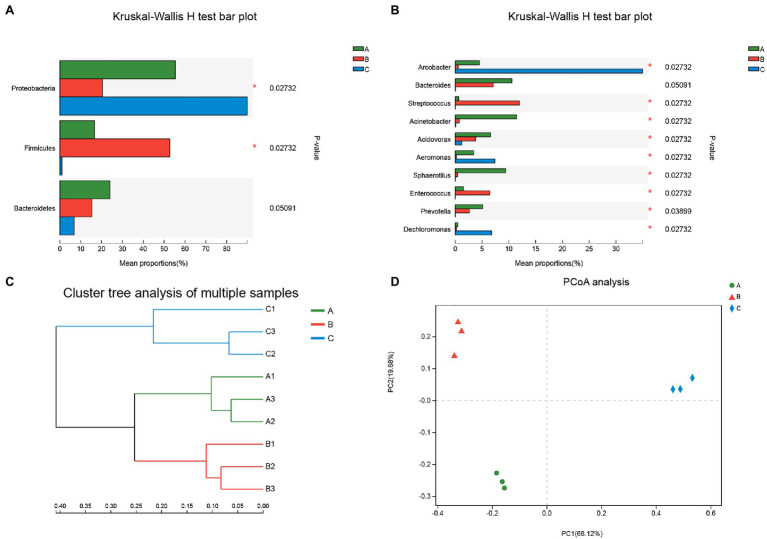
Statistical comparison of the relative abundance. **(A,B)** Microbiota composition difference in Phylum and Genus level; **(C,D)** Relationship of microbiota community between three groups analyzed on cluster tree and principal coordinate analysis (PCoA). Green (bar, line, or circle), A group, samples from Affiliated Hospital of Southwest Medical University; Red (bar, line, or triangle), B group, samples from Affiliated TCM Hospital of Southwest Medical University; Blur (bar, line, or diamond), C group, samples from Hospital of Stomatology Southwest Medical University. Differences were considered statistically significant at * p < 0.05 level.

To verify the differences in the bacterial community at the phylum and genus levels, the Kruskal-Wallis H test was used to analyze the main community abundance. The data are shown in Figure 2A. A significant number of sequences affiliated with Proteobacteria and Firmicutes were found in the wastewaters from the three hospitals (*p*<0.05; Figure 2A). The mean proportions differed significantly for nine genera (*p*<0.05; Figure 2B); only *Bacteroides* was not statistically significant. Notably, the composition of *Bacteroides* in the samples from all three hospitals was different, contributing 24.05% of the total microbiome in the A group, 15.43% in the B group, and 6.86% in the C group. According to the cluster tree analysis (Figure 2C), the microbiota composition structure was significantly different among the three groups, although, the A and B groups were more similar to each other compared to the C group. The PCoA separated the samples far from the central parallel axis 1 (PC1, 68.12%), indicating that the gene compositions of the wastewaters from these three hospitals also differed significantly (Figure 2D).

### Occurrence of ARGs in Hospital Wastewater Analyzed With ARDB

**Figure 3:**
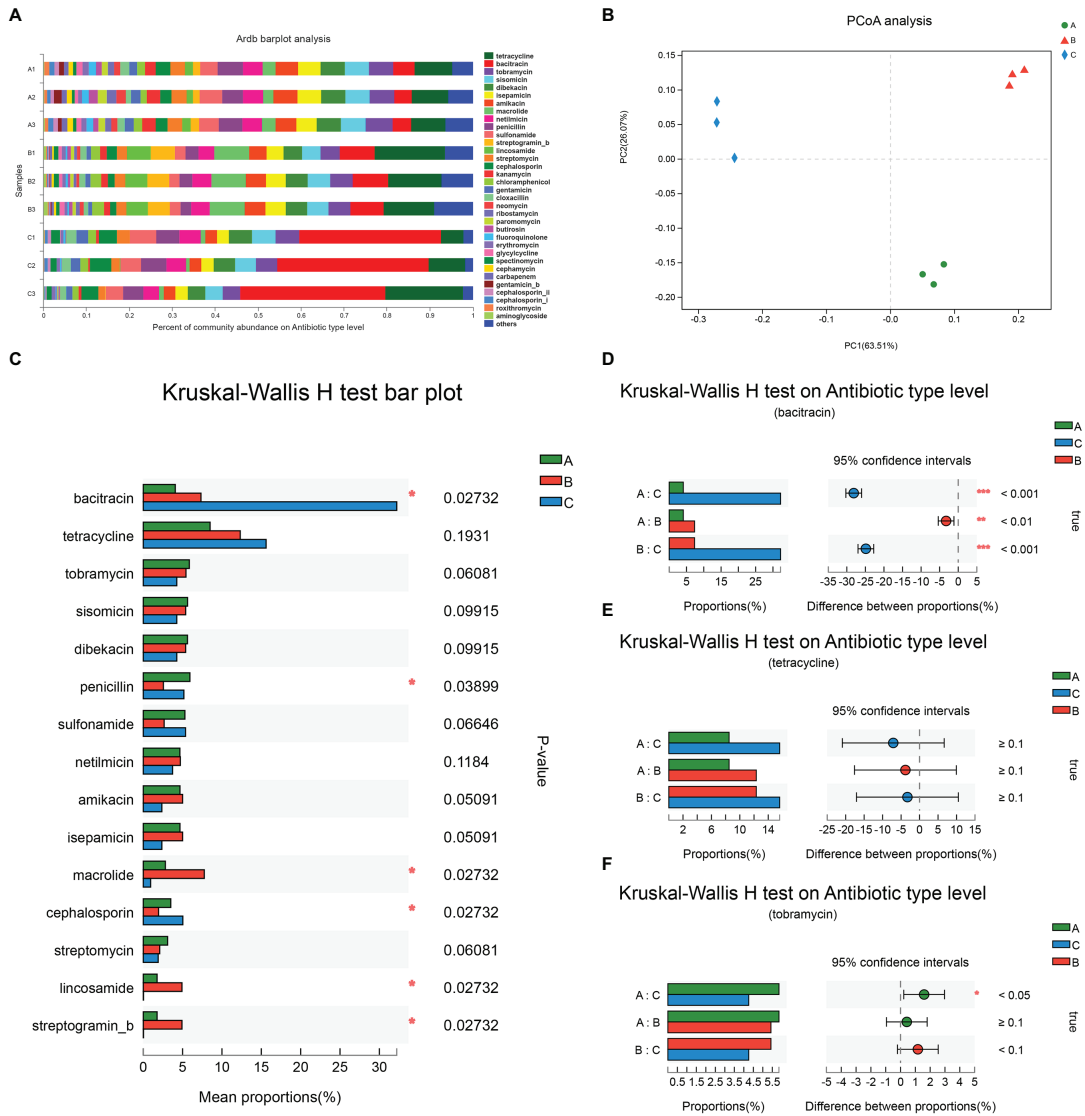
Community abundance of antibiotic resistance genes (ARGs) and statistical comparison on antibiotic type level. **(A)** Percentage of community abundance on antibiotic type level; **(B)** PCoA analysis of community on antibiotic type level; **(C)** The proportions of ARGs in three groups; **(D)** The composition difference of anti-bacitracin genes in pairwise comparison; **(E)** The composition difference of anti-tetracycline genes in pairwise comparison; and **(F)** The composition difference of anti-tobramycin genes in pairwise comparison. Differences were considered statistically significant at ^*^p<0.05; ^**^p<0.01; and ^***^p<0.001 level. A group, samples from Affiliated Hospital of Southwest Medical University; Red triangle, B group, samples from Affiliated TCM Hospital of Southwest Medical University; Blur diamond, C group, samples from Hospital of Stomatology Southwest Medical University.

Using the ARDB, 34 types of ARGs were detected in the wastewaters of the three hospitals wastewater. As shown in Figure 3A, ARGs associated with bacitracin were the most abundant resistance genes in the wastewaters from the A and B groups, while the ARGs associated with tetracycline were the most abundant in the wastewater of the C group. The community abundance of ARGs was significantly different in the PCoA analysis; the points of these three groups were located in different quadrants (Figure 3B). Depending on the type of ARGs, the diversity of ARGs in the C group was markedly lower than that in the other two groups. There were 438, 474, and 212 types of ARGs in the wastewaters of the A, B, and C groups, respectively.

To verify the community prevalence of general ARGs in hospital wastewater, we used the Kruskal-Wallis H test to analyze the composition of general ARGs. Of the 15 main composition types of ARGs, six types differed in the wastewaters of the three groups (Figure 3C; *p*<0.05). Among the antibiotic-relevant resistant genes, the three most abundant ARGs were associated with bacitracin, tetracycline, and tobramycin. The pairwise comparison of the three most abundant ARGs indicated differences between each two hospitals (Figures 3D–F), with the abundance of ARGs for bacitracin being statistically significant (*p*<0.01). There were no significant differences in the abundance of tetracycline resistance genes among the samples from the three hospitals. The percentage of ARGs associated with tobramycin differed between the A and C groups, while no significant difference was observed between the A and B or B and C groups.

### Occurrence of Total Genes of Bacteria Analyzed With Card

**Figure 4:**
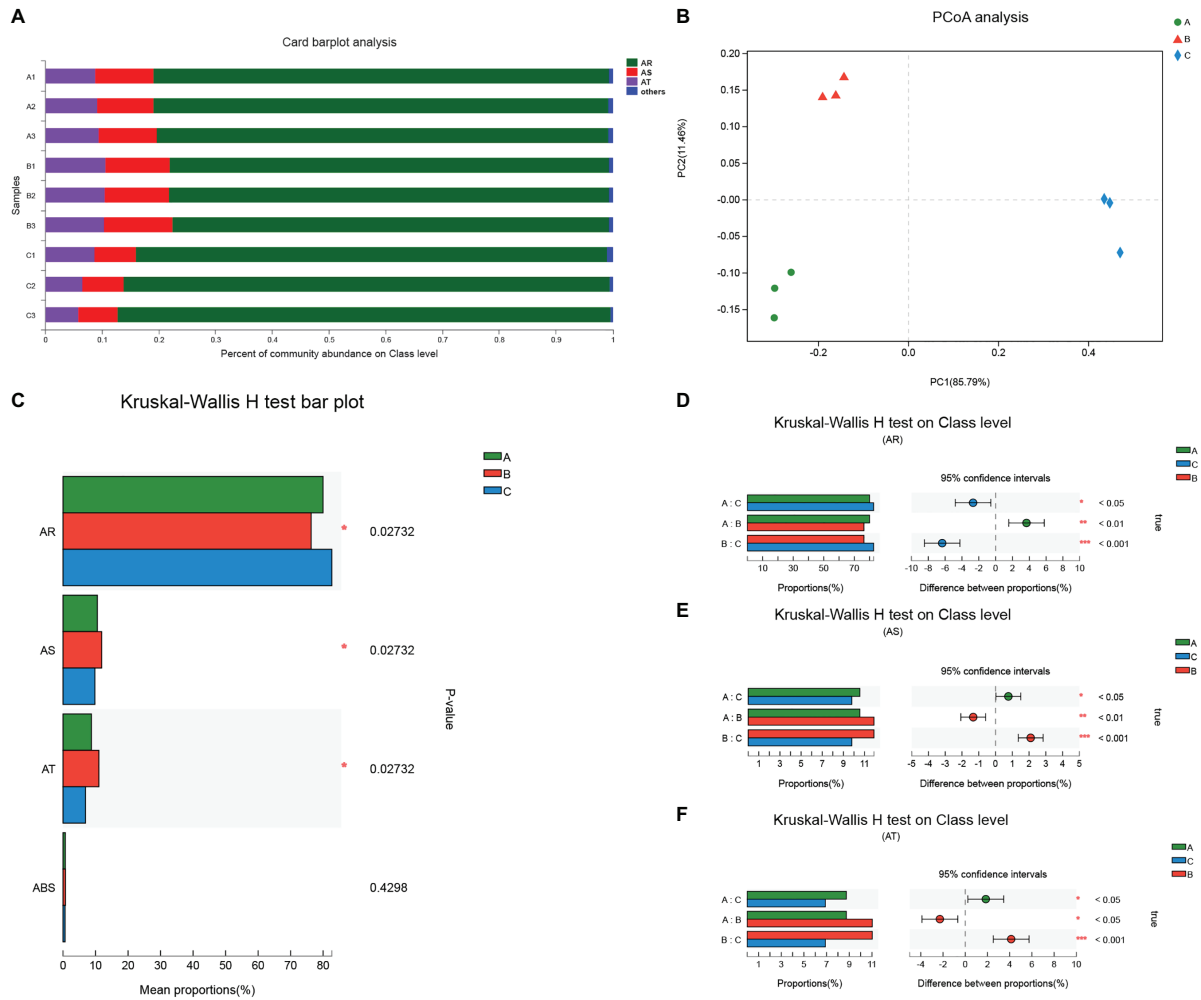
Community abundance of total ARGs and statistical comparison on class type level. **(A)** Percentage of community abundance on antibiotic class type level; **(B)** PCoA analysis of community on antibiotic type level; **(C)** The difference of proportions of ARGs on class level compared in three groups; **(D)** The composition difference of ARGs in pairwise comparison; **(E)** The composition difference of antibiotic sensitive (AS) genes in pairwise comparison; and **(F)** The composition difference of antibiotic target (AT) genes in pairwise comparison. Differences were considered statistically significant at ^*^p<0.05; ^**^p<0.01; and ^***^p<0.001 level.

The community abundance of bacterial comprehensive antibiotic resistance genes in the wastewater analyzed with CARD differed between the three groups (Figure 4A). The percentage of AR genes were the most prevalent class of genes among the wastewaters of the three hospitals, and the composition of antibiotic sensitive (AS) genes and antibiotic target (AT) genes varied. According to the PCoA of the bacterial community gene class type for the three groups, the points were located in different quadrants and far from the central parallel axis 1 (PC1, 85.79%; Figure 4B).

An in-depth analysis of the various class types was analyzed using the Kruskal-Wallis H test. As shown in Figure 4C, the proportion of AR, AS, and AT classes in the wastewaters from the three hospitals varied (*p*<0.05). The differences in comprehensive antibiotic resistance genes for each of the pairwise comparisons at AR, AS, and AT class are shown in Figures 4D–F.

### Correlation Analysis Between Species and ARGs Abundance

**Figure 5:**
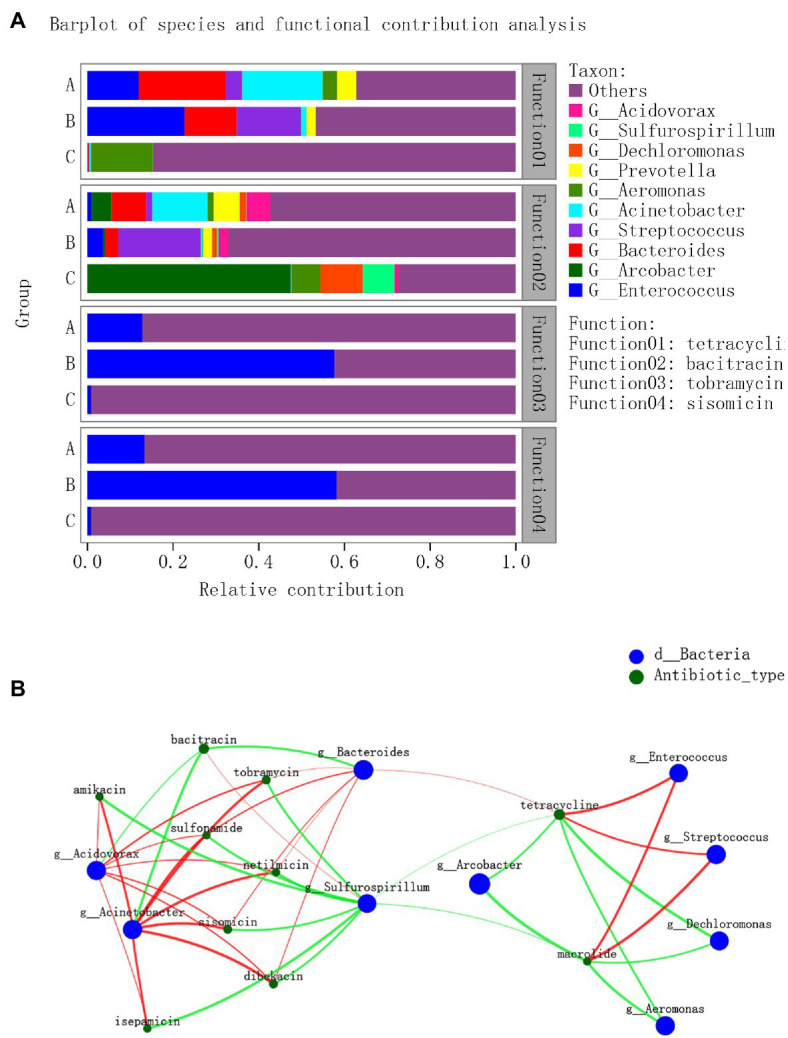
Correlations between species and corresponding ARGs. **(A)** Correlation of microbiota genus and ARGs relative antibiotics type; **(B)** Correlation of microbiota genus and comprehensive ARGs on antibiotic type level. The nodes were colored according to ARG types and genus. A connection represents a strong Spearman’s correlation coefficient (*ρ*>0.5) and significant (value of *p*<0.05) correlation. The color of the line indicates positive and negative correlation, red indicates positive correlation, and green indicates negative correlation.

The possible correlation between ARG types and bacterial genera was assessed under antibiotic type level. As shown in Figure 5A, *Aeromonas* contribute 14.50% for tetracycli ARG in C hospital wastewater, while *Enterococcus*, *Bacteroides*, *Streptococcus*, and *Acinetobacter* provided tetracycli ARG with different relative contributions in A and B groups. For bacitracin ARG, several types of bacteria were correlated with different relative abundances, while *Arcobacter* contributed with 47.49% in the C group hospital. *Enterococcus* contributed more than 50% for the tobramycin and sisomicin ARG type in B and C groups, while it contributed 12.80 and 13.31% for tobramycin and sisomicin ARG types in A and B groups, respectively; and they were both only 0.85% in C group. These results indicated that to reduce one type of ARGs in hospital wastewater, different types of bacterial species should be considered.

Then, a network analysis approach was applied to explore the correlation between ARG types and microbial taxa on antibiotic type level (Figure 5B). There were 10 antibiotic types ARGs co-occurring with nine genera of bacteria. *Acinetobacter* was the possible host positively associated with six types of ARGs: amikacin, isepamicin, sulforamide, tobramycin, sisomicin, and netilmicin. *Acidovorax* was positively correlated with seven types of ARGs: amikacin, tobramycin, sulfonamide, netilmicin, sisomicin, dibekacin, and isepamicin. In addition, there were positive correlations between six ARGs types and *Bacteroides*. *Enterococcus* and *Streptococcus* were found to be host of tetracycline and macrolide ARGs.

## Discussion

Hospital wastewater contains high concentrations of antibiotics and several types of pathogenic bacteria, which are the main source of antibiotic-resistant bacteria and ARGs (Hassoun-Kheir et al., 2020). In this study, we attempted to elucidate the differences in the composition of bacteria in wastewaters from three university-affiliated hospitals in Luzhou city, Sichuan Province, PR China, using high-throughput sequencing analysis. These three hospitals consisted of one general hospital, one general TCM hospital, and one stomatological hospital. By comparing the corresponding species and taxonomic annotation information with the NCBI NR database, our results showed that the community composition of bacteria in the wastewaters was different between hospitals at both the phyla and genera levels with significantly different communities of bacteria in each of them (Figures 1, 2). In the wastewater from three comprehensive hospitals with general departments, the community compositions of bacteria were more similar, *Arcobacter*, *Acinetobacter*, and *Dechloromonas* phyla were predominant with different relative abundances (Wang et al., 2018). The bacterial community composition of wastewater from the oncological hospital have shown a higher difference compared to the general hospital (Szekeres et al., 2017). The cluster tree and PCoA analysis for the relationship of community abundance between the three groups in this study also were similar to these results. The composition of bacteria in the wastewaters of the A and B groups was more similar to each other than to that of the C group. This may have occurred because both the A and B groups covered general medical practice, while the C group only focused on stomatology. Herein, relative protection should be applied as a measure to avoid the spread of infections according to the different bacterial compositions in the wastewater of the hospital. For example, *Arcobacter* was the main contributor to the wastewater from the C group; thus, more resources and attention should be focused on preventing infections and treating the wastewater specifically against *Arcobacter*. In addition, wastewater pretreatments can be targeted to harmful bacteria considering their properties. As Firmicutes live in an anaerobic environment and also are associated with high antibiotic and pollutant levels (Li et al., 2011; Bäumlisberger et al., 2015), monitoring the condition of wastewater may control the abundance of bacteria of this phylum.

Currently, most wastewaters are treated with sewage processing techniques before discharging into the urban sewage, which can remove some antibiotics resistance bacteria (Huang et al., 2012; Majeed et al., 2021). Yet, it still contains higher ARGs than natural water, and one of the main sources of ARGs is hospital effluent (Verlicchi et al., 2010). There were fewer types of ARGs in the wastewater from specialized hospitals compared with general hospitals (Szekeres et al., 2017). In this study, the ARGs in wastewaters from three hospitals were analyzed using the ARDB and CARD. As shown in Figure 3, the diversity of the ARGs was the lowest for the C group, however, the ARGs types and percentages in the wastewaters were different between the three groups. Among the 15 main types of ARGs, six types differed in the wastewaters of the three hospitals. For the common antibiotics (bacitracin, tetracycline, and tobramycin), there were significant differences in the levels of bacitracin ARGs among the three groups. In addition, the abundance of bacitracin-resistant bacteria in the C group was significantly more than that in the other two groups. Thus, disinfection and preventive measures used in hospitals that specialize in stomatology should target these specific bacteria.

For the total antibiotic resistance gene analysis using the CARD, the most common class of genes in wastewater from the three hospitals was AR, and together with the other two classes, AS and AT, their compositions were varied in three groups. This result is consistent with the findings of the bacterial community characteristic and ARG type analyses (Figures 2D, 3B).

In addition to the correlation between ARG types and bacteria genera, the results indicated that the same ARGs contributed to different bacterial genera with various relatives in the wastewater from three groups. For example, *Enterococcus*, *Bacteroides*, *Streptococcus*, and *Acinetobacter* contributed to tetracycli ARGs in A and B groups, while it comes from *Aeromonas* in the C group. This means that even for the treatment of the same resistance gene, the unique characteristics of species in wastewater should be considered according to different hospitals.

In conclusion, we used high-throughput sequencing to analyze bacterial community composition and ARGs in untreated wastewater samples collected from three university-affiliated hospitals. We found that significant differences in the bacterial community characteristics and ARG composition among the three hospitals and they differed between the types of hospital. Based on the differences in the bacterial communities and ARG compositions between the three types of hospitals, our results suggested that targeted prevention and control measures against related microbiota should be considered, and hospital wastewaters should be treated more specifically for pathogens that are present in it before the discharge into the urban sewage system. However, the wastewater samples were collected in the same season, which cannot cover the bacteria composition during the rest of the year, thus more samples from different seasons may give more information. Otherwise, there were no data about the antibiotic types in raw hospital wastewater, though it gave a negative correlation between the concentrations of antibiotics and ARGs (Wang et al., 2018). Another limitation of this study was that there was no physiochemical analysis for the raw wastewater, which may be affected by the seasons or other environmental conditions. This should be considered in future studies on hospital wastewater. Moreover, special techniques for preventing pathogen infection and release need to be identified according to the medical treatments being offered in a hospital.

## Data Availability Statement

The datasets presented in this study can be found in online repositories. The names of the repository/repositories and accession number(s) can be found at: https://www.ncbi.nlm.nih.gov/, PRJNA723368.

## Author Contributions

CS conceived and designed the experiments. NT collected and processed the samples from the hospital wastewater system. CS and XG analyzed the metagenomics sequence data, created the figures, and wrote the manuscript. HL and LL helped to plan the project and contributed to development of the manuscript. QF and QZ assisted with sample collection and DNA extraction. All authors contributed to the article and approved the submitted version.

## Conflict of Interest

The authors declare that the research was conducted in the absence of any commercial or financial relationships that could be construed as a potential conflict of interest.

## Publisher’s Note

All claims expressed in this article are solely those of the authors and do not necessarily represent those of their affiliated organizations, or those of the publisher, the editors and the reviewers. Any product that may be evaluated in this article, or claim that may be made by its manufacturer, is not guaranteed or endorsed by the publisher.

## Footnotes

^1^ https://github.com/jstjohn/SeqPrep

^2^ https://github.com/najoshi/sickle

^3^ https://github.com/voutcn/megahit

^5^ http://www.ncbi.nlm.nih.gov/Taxonomy/taxonomyhome.html/index.cgi?chapter=tgencodes#SG1

^6^ https://github.com/ShujiaHuang/SOAPaligner

^7^ http://blast.ncbi.nlm.nih.gov/Blast.cgi

^8^ https://www.genome.jp/kegg/

^9^ http://ardb.cbcb.umd.edu/

^10^ https://card.mcmaster.ca/

^11^ https://cloud.majorbio.com/
