# Supplementary material for: Metagenomic Analysis of Antibiotic Resistance Genes in Untreated Wastewater From Three Different Hospitals
Source: Front Microbiol. 2021 Aug 24;12:709051. doi: 10.3389/fmicb.2021.709051 (PMC8421800; doi:10.3389/fmicb.2021.709051)
Supplement: Supplementary file 1 [file Table_1.DOCX]

**Supplementary Materials:**

**Metagenomic analysis of antibiotic resistance in untreated wastewater from three different hospitals**

Xiurong Guo^1,#^, Nan Tang^1,#^, Hui Lei^1^, Qi Fang^1^, Li Liu^1^, Quan Zhou^1^, Can Song^1,^*

1. School of Pharmacy, Southwest Medical University, Luzhou, 646000, Sichuan, China

* Corresponding: cansong@swmu.edu.cn (Can Song).

# These authors contributed equally to this work.

| **Samples** | **Clean reads** | **Clean base(bp)** | **Percent in raw reads(%)** | **Percent in raw bases(%)** |
| --- | --- | --- | --- | --- |
| **A1** | 97000964 | 14596792272 | 98.20517749 | 97.86757219 |
| **A2** | 88492558 | 13334654719 | 98.30449914 | 98.10055697 |
| **A3** | 89608968 | 13491728293 | 97.39824033 | 97.11588543 |
| **B1** | 90342758 | 13608151438 | 97.96340154 | 97.72207909 |
| **B2** | 89163642 | 13421446243 | 98.36318259 | 98.05441242 |
| **B3** | 99810324 | 15024440149 | 98.18109319 | 97.8754448 |
| **C1** | 109550546 | 16490722469 | 98.00615062 | 97.70156511 |
| **C2** | 94045656 | 14147056790 | 98.18681861 | 97.81458079 |
| **C3** | 89353196 | 13455739249 | 98.43900166 | 98.17201947 |

Table S1. Summary for the metagenomic clean sequencing.

Table S2. Summary for the metagenomic assembly, annotation and ORF prediction.

| Sample | ORFs | Total Length(bp) | Average Length(bp) | Max(bp) | Min(bp) |
| --- | --- | --- | --- | --- | --- |
| A1 | 1156344 | 533256377 | 461.16 | 15386 | 100 |
| A2 | 1183634 | 562722937 | 475.42 | 10665 | 100 |
| A3 | 1178521 | 550335185 | 466.97 | 15787 | 100 |
| B1 | 1416160 | 677563931 | 478.45 | 16887 | 100 |
| B2 | 1393635 | 660110829 | 473.66 | 18246 | 100 |
| B3 | 1443920 | 669723155 | 463.82 | 15091 | 100 |
| C1 | 240054 | 135624035 | 564.97 | 23421 | 100 |
| C2 | 213729 | 115282820 | 539.39 | 32412 | 100 |
| C3 | 199385 | 112817442 | 565.83 | 18301 | 100 |

| Genes | Total length (bp) | Average length (bp) | Catalog genes | Catalog total length (bp) | Catalog average length (bp) |
| --- | --- | --- | --- | --- | --- |
| 8425382 | 4017436711 | 476.83 | 2677368 | 1473029971 | 550.18 |

Table S3. Summary for the metagenomic non-redundant gene catalog.
